# Polyaromatic hydrocarbons in pollution: a heart‐breaking matter

**DOI:** 10.1113/JP278885

**Published:** 2020-01-15

**Authors:** C. R. Marris, S. N. Kompella, M. R. Miller, J. P. Incardona, F. Brette, J. C. Hancox, E. Sørhus, H. A. Shiels

**Affiliations:** ^1^ Division of Cardiovascular Sciences Faculty of Biology Medicine and Health University of Manchester Manchester UK; ^2^ BHF Centre for Cardiovascular Science Queens Medical Research Institute The University of Edinburgh Edinburgh UK; ^3^ Environmental and Fisheries Sciences Division Northwest Fisheries Science Center National Oceanic and Atmospheric Administration Seattle WA 98112 USA; ^4^ INSERM Centre de Recherche Cardio‐Thoracique de Bordeaux U1045 Bordeaux France; ^5^ Université de Bordeaux Centre de Recherche Cardio‐Thoracique U1045 Bordeaux France; ^6^ IHU Liryc Electrophysiology and Heart Modeling Institute Fondation Bordeaux Université Pessac‐Bordeaux France; ^7^ School of Physiology Pharmacology and Neuroscience Bristol Heart Institute University of Bristol Bristol BS2 8HW UK; ^8^ Institute of Marine Research PO Box 1870 Nordes NO‐5871 Bergen Norway

**Keywords:** air pollution, cardiotoxicity, cardiovascular dysfunction, heart disease, oil spills, PAH, phenanthrene, particulate matter, PM, PM_2.5_

## Abstract

Air pollution is associated with detrimental effects on human health, including decreased cardiovascular function. However, the causative mechanisms behind these effects have yet to be fully elucidated. Here we review the current epidemiological, clinical and experimental evidence linking pollution with cardiovascular dysfunction. Our focus is on particulate matter (PM) and the associated low molecular weight polycyclic aromatic hydrocarbons (PAHs) as key mediators of cardiotoxicity. We begin by reviewing the growing epidemiological evidence linking air pollution to cardiovascular dysfunction in humans. We next address the pollution‐based cardiotoxic mechanisms first identified in fish following the release of large quantities of PAHs into the marine environment from point oil spills (e.g. Deepwater Horizon). We finish by discussing the current state of mechanistic knowledge linking PM and PAH exposure to mammalian cardiovascular patho‐physiologies such as atherosclerosis, cardiac hypertrophy, arrhythmias, contractile dysfunction and the underlying alterations in gene regulation. Our aim is to show conservation of toxicant pathways and cellular targets across vertebrate hearts to allow a broad framework of the global problem of cardiotoxic pollution to be established. AhR; Aryl hydrocarbon receptor. Dark lines indicate topics discussed in this review. Grey lines indicate topics reviewed elsewhere.

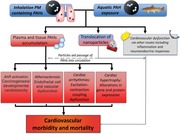

## Graphical abstract

Polycyclic aromatic hydrocarbons are common in both air and aquatic pollution. Exposure to these pollutants can lead to PAH accumulation in the body and cause cardiovascular dysfunction via a number of direct and indirect mechanisms. PAH exposure is associated with arrhythmias, hypertrophy, atherosclerosis and developmental toxicity (among others). This review discusses current evidence linking PAH‐based pollution and cardiovascular disease in vertebrates.

**Abbreviations**
AA: arachidonic acid
AhR: aryl hydrocarbon receptor
AP: action potential
APD: Action potential duration
ANP: atrial natriuretic protein
BaP: Benzo[a]pyrene
BMP10: bone morphogenetic protein 10
BNP: brain natriuretic protein
CdC42: cell division control protein 42 homolog
CREB: cAMP response element‐binding protein
CVD/CVS: cardiovascular disease/cardiovascular system
CYP1A1/1B1: cytochrome P450 1A1/1B1
DCM: dilated cardiomyopathy
DEP: diesel exhaust particles
DWH: deepwater horizon
EC: excitation‐contraction
ERG: Ether‐à‐go‐go‐related gene K^+^ channel
HCAEC: human coronary artery endothelial cells
HUVEC: human umbilical vascular endothelial cells
hiPSC: human induced pluripotent stem cells
IUPS: international union of physiological sciences
LTCC: L‐type calcium channels
MMP‐9: matrix metalloproteinase‐9
NFAT: nuclear factor of activated T‐cells
PAH: polycyclic aromatic hydrocarbons
Phe: phenanthrene
PLA_2_: phospholipase A_2_
PM: particulate matter
ROFA: residual oil fly ash
SERCA: sarcoplasmic reticulum calcium ATPase
SR: sarcoplasmic reticulum
Tbx5: T‐box 5
TCDD: 2,3,7,8‐Tetrachlorodibenzo‐*p*‐dioxin
TGF‐β: transforming growth factor‐β

## Introduction

The disease burden from ambient air pollution is becoming increasingly apparent, with a host of recent studies drawing close association between pollution levels and reduced life expectancy (Hoek *et al*. 2013; Lelieveld *et al*. 2019). An estimated 4.2 million premature human deaths worldwide are attributed to ambient air pollution (World Health Organization (WHO), 2018). Over 90% of the population live in areas where air pollution is above the World Health Organization's guidelines and, with air pollution levels still on the rise in many countries, this threat is ever growing (WHO, 2018). Air pollution is derived from a vast range of sources, but combustion of fossil fuels represents a significant proportion of the pollutants known to be detrimental to health. In parallel, industrial pollutant discharge and natural oil seeps in aquatic ecosystems, together with observations from disastrous oil spills, have demonstrated the considerable damage that other forms of fossil fuel pollution can impose on both aquatic life and human livelihood (Smith & Levy, 1990; Beyer *et al*. 1998; Naes & Oug, 1998; Zakaria *et al*. 2002; Pampanin & Sydnes, 2013; Stogiannidis & Laane, 2015).

What is becoming increasingly evident is the detrimental effect of fossil fuel‐derived pollution on the cardiovascular system (CVS). Despite the lung being the major entry point into the body and thus the first target of exposure, numerous epidemiological studies have shown strong correlations between air pollution and cardiovascular diseases (CVDs) (Shah *et al*. 2013; Franklin *et al*. 2015). Indeed, mortality attributed to air pollution's impact on CVD outweighs mortality due to impact on respiratory diseases (Cohen *et al*. 2017).

In fish, the cardiotoxicity of exposure to fossil fuels has been recognised for several decades. The negative impact of crude oil exposure on cardiac function was clearly identified in the years following the 1989 Exxon Valdez oil spill (Incardona *et al*. 2009), and a deeper mechanistic understanding of the cardiotoxic pathways followed the 2010 Deepwater Horizon (DWH) blowout (Brette *et al*. 2014, 2017). While there are undoubtedly clear differences in air pollutants and aquatic environmental pollution (e.g. physicochemical properties of the pollutants, interaction between pollutants and the environment, the biology of the species exposed and the route of exposure), it has become apparent that parallels exist, especially in terms of the ability of these pollutants to cause cardiovascular toxicity. This review builds on a focus session at the 2017 International Union of Physiological Sciences (IUPS) World Congress (http://www.iups.org/) to outline the current epidemiological, clinical and experimental evidence by which pollution can impact on the heart and other aspects of the CVS. Our focus is on the cardiotoxicity of the lower molecular weight polycyclic aromatic hydrocarbons (PAHs) as key mediators of these effects. This review outlines (1) the growing epidemiological evidence linking air pollution to cardiovascular dysfunction, (2) the importance of particulate matter (PM) and PAHs in cardiotoxicity, (3) the key mechanisms of cardiotoxicity identified in the flurry of fish heart research that followed the Exxon Valdez and DWH oil spills, and (4) the current state of mechanistic knowledge linking PAH exposure to cardiovascular patho‐physiologies such as atherosclerosis, cardiac hypertrophy, arrhythmias, contractile dysfunction and the underlying alterations in gene regulation. In this review we draw on studies from humans, other mammals and fish which collectively form our current understanding of pollution‐based cardiovascular toxicity. In the final section we discuss the implications of interspecies diversity for extrapolating risks identified in aquatic pollution to those of human exposure to air pollution.

## Airborne particulate matter and human health risks

**Figure 1:**
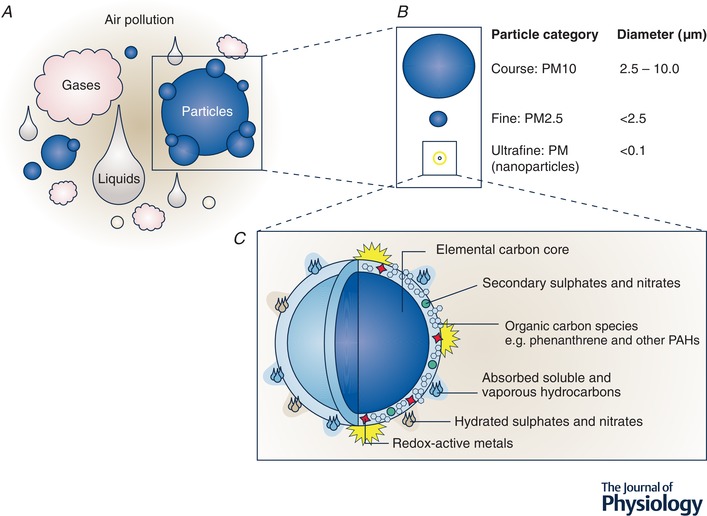
Constituents of air pollution *A*, air pollution can be broadly characterized into gases, liquids and particles. PAHs can be found in the gaseous phase of air pollution as well as binding to the particle surface. *B*, particles can be further classified into size categories of particulate matter (PM). For perspective, if a human hair has a width of approximately 50 µm (0.05 mm) then PM_10_ encompasses particles with a diameter below a fifth of this size (<10 µm). Fine PM (<2.5 µm) would be a quarter of this size, and smaller still, ultrafine (nano) particles a hundredth of the size of PM_10_ (<0.1 µm). *C*, schematic diagram providing an example of the complex composition of a combustion derived ultrafine (nano) particle, such as a diesel exhaust particle (DEP), a common PM in urban air. The carbon core of DEP is coated with a diverse range of chemical species including reactive transition metals and polyaromatic hydrocarbons like phenanthrene. Detail of surface chemicals in *C* is not to scale. Figure reproduced from *Environmental Health Perspectives* with permission from the authors (Stone *et al*. 2017).

Numerous epidemiological studies have linked air pollution to cardiovascular morbidity and mortality. Airborne PM is a key pollutant (in terms of strength, and in many cases magnitude, of associations) driving the cardiovascular effects. PM exposure is linked to cardiac arrhythmias, alterations in heart rate variability, myocardial infarction, arterial vasoconstriction, increased blood coagulability, atherosclerosis (vascular plaques), heart failure and stroke (Mills *et al*. 2009; Brook *et al*. 2010; Brook & Brook, 2011; Shah *et al*. 2013; Franklin *et al*. 2015; Newby *et al*. 2015). Atmospheric PM encompasses a complex mixture of particulates and liquid droplets with a wide‐ranging chemical composition (see Fig. 1; Niemann *et al*. 2017; Environmental Protection Agency, 2018). PM is grouped into three classes based on particle size (Fig. 1B). Course particles (with a diameter between 2.5 and 10 µm) tend to be mechanically derived from construction and demolition, mining, agriculture, tyre fragmentation and other road dust. Fine (diameter of ≤2.5 µm; PM_2.5_) and ultrafine particles (also known as nanoparticles; diameter ≤ 0.1 µm) have both natural and anthropogenic sources. Ultrafine PM and a substantial proportion of PM_2.5_, are derived from combustive sources such as motor vehicle exhaust, industrial burning of coal and fuel oil, cigarette smoke and residential wood burning. PM_2.5_ is widely measured in the environment as an indicator of air quality. This class of PM is particularly concerning due to the high penetration deep into the lungs after inhalation (Lee *et al*. 2018; and see below) and for the large reactive surface area of these small particles. Ultrafine PM cannot presently be routinely measured in the environment, yet has the potential to be an even greater threat to health due to the markedly greater surface area these particles have and that they may gain access (i.e. translocate) to other organs of the body (Donaldson *et al*. 2013).

Positive associations between PM_2.5_ levels and incidences of stroke are evident in areas with increased industrialization such as India, China and Africa (Shah *et al*. 2015; Lee *et al*. 2018). Two long‐term studies based in the USA in 2007 (Miller *et al*. 2007) and 2011 (Lipsett *et al*. 2011), identified a significant increase in stroke incidence for every 10 µg m^−3^ increase in PM_2.5_ levels (35% and 19%, respectively). Similarly, a ∼20% increase in stroke incidence has been observed in various European countries which met their air quality standards, indicating that current standards may not be sufficient (Lee *et al*. 2018). Concern for current air quality standards was bolstered by Aung *et al*. (2018), who showed correlations between UK addresses, ambient air quality data (at levels within current UK air quality standards) and biventricular dilatation that is suggestive of detrimental cardiac remodelling.

Short‐term exposure to air pollution has also been associated with severe cardiac complications. A study in the Greater Boston area found a strong association between the onset of ischaemic stroke and traffic‐related pollutant exposure 12—14 h prior (Wellenius *et al*. 2012). Associations have even been found between the onset of myocardial infarctions 2 h after high PM exposure associated with traffic (Peters *et al*. 2001). Daily variations in pollutant levels are also associated with increases in hospital admissions for ischaemic heart disease, particularly in vulnerable subgroups like those with accompanying congestive heart failure and arrhythmias (Mann *et al*. 2002). It is possible that other vulnerable groups (e.g. newborns, children, the elderly and immunocompromised individuals) may also be particularly susceptible to the impacts of air pollution on the CVS but epidemiological studies are lacking and calls for greater focus in this area have been made (Sram *et al*. 2013; Wittkopp *et al*. 2016).

There is growing recognition of the effects of indoor air pollution on health (Lim *et al*. 2012; WHO, 2014). Indoor air pollution has arguably a greater diversity of sources (e.g. heating, cooking, smoking, moulds, pet dander, mosquito coils, chemical aerosols, cleaning products, ingress of outdoor air pollution, and many others), with marked differences between developed and developing nations. Similar to outdoor/ambient pollution, PM is the pollutant of concern, showing strong associations with CVDs (Mitter *et al*. 2016). With people spending more than 80% of their time indoors, and with the ready use of unclean/inappropriate fuel sources in developing nations, the true scale of indoor air pollution is likely to come to prominence in the next decade as research in this area grows.

Already, the potential impact of pollution has been substantiated, with Lelieveld et al. recently estimating a new total excess mortality rate of 659,000 per year due to air pollution in the 28 countries of the European Union (Lelieveld *et al*. 2019), a vast increase from the 400,000 per year currently acknowledged by the European Environment Agency (Agency, 2019).

## Inhaled particles and the cardiovascular system

Despite the considerable evidence linking air pollution and CVD, the biological mechanisms linking this association are not fully elucidated. The particulate constituents of air pollution appear to drive the cardiovascular actions. Deposition of PM in the lungs depends on particle size (United States Environmental Protection Agency, 2017). Course particles are largely deposited in the upper airways and are then cleared by ciliary action. Fine and ultrafine particles can reach the lower regions of the lung: the acini, consisting of respiratory bronchioles, alveolar ducts and alveoli where gas exchange occurs. Following deposition on the alveolar walls, fine particles can be cleared with the assistance of alveolar macrophages; however, a proportion of particulates (especially smaller particles) may evade capture or may trigger an inflammatory response within the lungs (Stone *et al*. 2017). Induction of inflammation with release of inflammatory mediators and activation of sensory afferents are likely to be means by which inhaled PM can induce cardiovascular effects; the former by passage of inflammatory or oxidative mediators into blood, and the latter by changes in autonomic function and neuroendocrine systems (Miller, 2014). Moreover, urban PM and diesel exhaust particles (DEP; a rich source of combustion‐derived nanoparticles in ambient air pollution, see Fig. 1*C*) can directly generate free radicals from their surface, and through activation of cellular enzymes induce oxidative stress and inflammation (Donaldson *et al*. 2005; Miller *et al*. 2009; Langrish *et al*. 2012). Both processes are known to impair vascular function in (otherwise) healthy blood vessels to promote disease (Münzel *et al*. 2018).

The smallest ultrafine nanoparticles (Fig. 1*B* and *C*) appear to be able to pass the alveolar barrier, enter the pulmonary circulation, and be carried to other regions of the body. Due to the very small numbers of ultrafine particles likely to translocate and the difficulty in detecting these particles systemically, it has been challenging to conclusively demonstrate that environmental ultrafine nanoparticles translocate in humans (Nemmar *et al*. 2002; Mills *et al*. 2006; Möller *et al*. 2008). However, experimental studies using model nanoparticles such as radiolabelled carbon or gold nanoparticles have robustly demonstrated that this pathway occurs in rodents (Geiser & Kreyling, 2010) and now recently in humans (Miller *et al*. 2017a,*b*). The translocation pathway is of importance as it provides a biological basis that could account for the widespread effects of inhaled PM across the CVS, and elsewhere in the body (Raftis & Miller, 2019). Thus while changes in autonomic function are likely to play a major role in the arrhythmogenic action of PM (Robertson *et al*. 2014; Buteau & Goldberg, 2016) and systemic inflammation will most likely exacerbate existing disease processes, the entry of PM into the systemic circulation expands the diversity of routes, targets and time course by which inhaled PM deregulates cardiovascular homeostasis. Access of particles to the blood also provides a means by which inhaled PM and its constituent surface chemicals can directly interact with the CVS.

## PAHs in air pollution

Combustion‐derived PM is predominantly carbon based; however, it has a complex composition. A central particle core is composed of both organic and elemental carbon (as well as other substances such as sulphates and nitrates). However, the particle surface is coated with a range of different chemical entities that include organic carbon species such as PAHs, as well as reactive metal species (e.g. iron, copper, nickel; Fig. 1*C*). The chemicals within this surface mixture, in particular the PAHs, are believed to greatly influence PM biological activity, and recent evidence links the cardiovascular toxicity of PM_2.5_ to its organic carbon component (Miller *et al*. 2012) in humans (Mills *et al*. 2011).

PAHs are a family of ubiquitous contaminants which can bind to particulates or can form small airborne particles in the gaseous phase of air pollution themselves. Studies in animals show exposure to DEP, but not ultrafine polystyrene particles, caused a pronounced pulmonary inflammatory response (Nemmar *et al*. 2004). Exposure to residual oil fly ash (ROFA) but not ‘clean’ black carbon, produced electrocardiographic changes (Wellenius *et al*. 2002), indicating that simply the physical presence of a (relatively inert) particle core is insufficient for a cardiovascular pathophysiological response. These studies are consistent with the bioavailability of PAHs in PM and in vehicle emissions. Indeed, respiratory epithelial cells exposed to PM_2.5_ take up PAHs (Penn *et al*. 2005), and in the case of the smallest sized particles in PM_2.5_, these may be transported across the alveolar epithelium and into the systemic circulation un‐metabolized (Gerde *et al*. 2001). The transport of PAHs to the heart from the pulmonary circulation would occur before significant hepatic metabolism (Incardona *et al*. 2005).

### Phenanthrene

**Figure 2:**
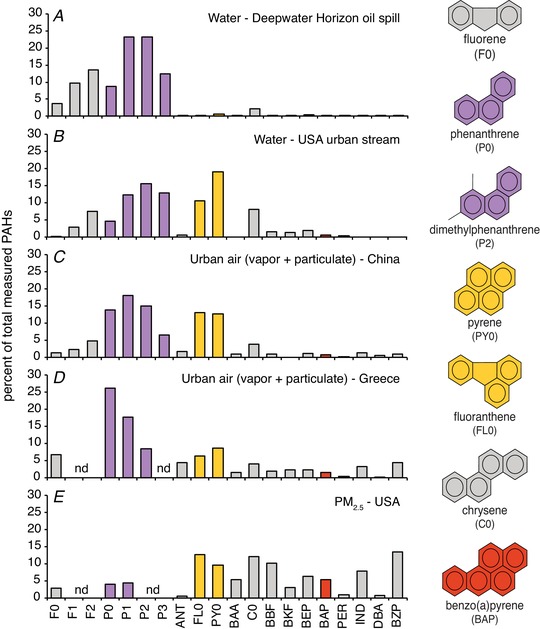
Comparison of PAH composition between water and air samples Composition profiles represent the percentage of the indicated PAHs normalized to the sum total of the measured set, with ring number and molecular weight increasing to the right. Parent non‐alkylated compounds are denoted with a 0 (e.g. F0, P0), while alkylated compounds (e.g. those with 1 or 2 additional methyl groups) are indicated by F1, F2, P1, P2, etc. Selected individual structures are shown on the right, with phenanthrene and its alkylated homologues indicated in purple, the 4‐ringed pyrogenic compounds indicated in gold (pyrene and fluoranthene), and the archetypal carcinogenic 5‐ringed PAH benzo(a)pyrene indicated in red. Five sample sources are shown. *A*, water from the area of the Gulf of Mexico impacted by the 2010 Deepwater Horizon oil spill (Incardona *et al*. 2014); *B*, passive samplers placed in an urban stream in Seattle (USA) that receives large quantities of stormwater runoff from nearby roadways (Scholz *et al*. 2011; J. P. Incardona, unpublished); *C*, a total high volume air sample from Guangzhou (China) collected July 2001 by combined glass fibre filter (GFF) and polyurethane foam (PUF) plug (Bi *et al*. 2003); *D*, the mean of 16 total GFF/PUF samples collected between November 2000 and February 2002 in Heraklion (Greece) (Tsapakis & Stephanou, 2005); *E*, mean of quarterly samples collected over the year 2004 using a PM_2.5_ particle composition monitoring system at a heavily trafficked urban site in Atlanta, USA (Li *et al*. 2009). Abbreviations: F, fluorene; P, phenanthrene; ANT, anthracene; FL, fluoranthene; PY, pyrene; BAA, benz(a)anthracene; C, chrysene; BBF, benzo(b)fluoranthene; BKF, benzo(k)fluoranthene; BEP, benzo(e)pyrene; BAP, benzo(a)pyrene; PER, perylene; IND, indeno(123‐cd)pyrene; DBA, dibenzanthracene; BZP, benzo(ghi)perylene; nd, not determined.

Each PAH compound contains two or more fused benzene rings (see right hand column in Fig. 2). There are two main sources of PAHs: (1) pyrogenic sources which involve anoxic or low oxygen combustion of fossil fuels and are dominated by the presence of larger 4–6 ring PAHs, and (2) petrogenic sources that include crude oils and associated by‐products and consist primarily of 2–3 ring PAHs. Tricyclic (3‐ringed) PAHs, such as phenanthrene (Phe) and its alkylated homologues (e.g. dimethylphenanthrene; Fig. 2), are naturally enriched in crude oil and refined fuels and they are present in most types of combustive emissions in large quantities (Fig. 2). Excluding 2‐ringed naphthalene, phenanthrenes are the most abundant PAH family in aquatic environments impacted by either oil spills or by urban (non‐point source) pollution (Fig. 2*A* and *B*), and in urban air (Fig. 2*C* and *D*) (Liu *et al*. 2001; Naumova *et al*. 2002; Bi *et al*. 2003; Tsapakis & Stephanou, 2005; Li *et al*. 2009; Scholz *et al*. 2011; Incardona *et al*. 2014). PM contains Phe (Abdel‐Shafy & Mansour, 2016) and significant levels of Phe can be found in the gaseous phase of vehicle exhaust (Schauer *et al*. 1999, 2002; Möllmann *et al*. 2006). Levels of Phe in London air were reported at 76–82 ng m^−3^(Halsall *et al*. 1994) and ∼10–150 nmol L^−1^ in the water of the Jiulong River Estuary and Western Xiamen Sea in China (Maskaoui *et al*. 2002). Therefore, terrestrial animals (including humans) and fishes come into contact with Phe through diet, drinking, breathing, smoking and through other routes of environmental exposure (Laurent *et al*. 2001; Grova *et al*. 2005; Zhong *et al*. 2011). Phe is known to exert direct toxicity in dogs where aerosolized Phe was rapidly transported into the bloodstream following inhalation (Gerde *et al*. 1993). Particle translocation may also assist the passage of Phe within PM to areas of susceptibility, given that translocated nanoparticles have been shown to preferentially accumulate at sites of vascular disease (Miller *et al*. 2017a).

While data on human plasma levels of PAHs are sparse, plasma concentrations of Phe and/or other PAH molecules can be found at the nanomolar level in animals (Zhong *et al*. 2011; Camacho *et al*. 2012). A recent study of maternal blood of pregnant (non‐smoker) women and cord blood of newborns reported Phe concentrations of 37.61 and 35.32 µg L^−1^, respectively; a concentration approximating to 200 nM (Scholz *et al*. 2011). It is important to note that Phe, like most tricyclic PAHs, is highly lipophilic with a logP (octanol/water) coefficient of ∼4.4, indicating tissue accumulation of Phe at levels higher than those observed in plasma (Carls *et al*. 1999; Heintz *et al*. 1999). This was notably shown in studies where tissue concentrations approximating to 3 µM were reported (Jacob & Seidel, 2002; Dhananjayan & Muralidharan, 2013), with levels being exacerbated by smoking (363 ng Phe per cigarette; Severson *et al*. 1976; Howard *et al*. 1998).

### The aryl hydrocarbon receptor (AhR) and the unique cardiotoxicity of low molecular weight PAHs

For many years the toxicology of PAHs was focused almost exclusively on a single 5‐ringed compound, benzo(a)pyrene (BaP). A dramatic increase in the prevalence of skin cancers among coal‐tar workers during the Industrial Revolution led to the identification of BaP as a metabolically activated chemical carcinogen (Phillips, 1983). Subsequent research showed that BaP activates an orphan transcription factor later described as the aryl hydrocarbon receptor (AhR) (Denison *et al*. 1998). AhR in turn regulates genes involved in PAH metabolism (Nebert *et al*. 2004). When activated, AhR migrates into the nucleus and binds to a specific sequence within xenobiotic responsive elements resulting in expression of xenobiotic metabolising enzymes, including cytochrome P450 1A1 (CYP1A1) and cytochrome P450 1B1 (CYP1B1) (Germolec *et al*. 1996; Korashy & El‐Kadi, 2006). CYPs have the ability to metabolize PAHs such as BaP into reactive metabolites which can alkylate macromolecules and have carcinogenic properties (Denison *et al*. 1988; Korashy & El‐Kadi, 2006). Interestingly, particles such as 2,3,7,8‐tetrachlorodibenzo‐*p‐*dioxin (TCDD) which are produced by organic/diesel combustion also interact with CYP pathways, and in mammalian embryos can increase mortality rates and cause severe cardiac hypertrophy (Walker *et al*. 1997; Dalton *et al*. 2001; Ivnitski *et al*. 2001; Lin *et al*. 2001; Kanzawa *et al*. 2004). Chronic exposure to TCDD in adults can also increase the incidence of spontaneous cardiomyopathy (Jokinen *et al*. 2003).

In developing fish, the larger 5‐ringed PAHs cause developmental cardiotoxicity in a manner similar to the very potent AhR ligands of the dioxin family (Incardona, 2017). With these compounds, toxicity is not linked to CYP1A‐mediated metabolism, but results from inappropriate AhR activation within developing cardiomyocytes, leading to reduced cardiomyocyte proliferation and heart malformation (for review, see Incardona, 2017). However, BaP and similar compounds make up a lower proportion of the total PAHs in water and air pollution (see Fig. 2).

Low molecular weight PAHs such as tricyclic Phe have been found to be weak AhR agonists with little to no carcinogenic activity (Barron *et al*. 2004). Thus, these chemical species were largely ignored through nearly eight decades of PAH research. However, more recent studies from fish indicate that not all PAH toxicity can be attributed to AhR activation (Incardona *et al*. 2005). In particular, and as detailed further below, Phe and complex PAH mixtures found in air and water pollution cause a functional cardiotoxicity that is *independent* of the AhR/CYP1A pathway and independent of cardiac developmental abnormalities.

### Cardiotoxicity of PAHs in fish

The toxicity of tricyclic (3‐ringed) PAHs became apparent from the effects of the 1989 Exxon Valdez oil spill on Pacific herring (*Clupea pallasi*) and pink salmon (*Oncorhynchus gorbuscha*). At the time of the spill, these fish were the basis of the largest commercial fisheries in Alaska, and their near‐shore spawning habitats were contaminated by oil. Field studies identified a malformation syndrome in herring and salmon embryos and larvae that was linked to oiled shoreline exposure. Subsequent laboratory studies associated this syndrome with the uptake of 3‐ringed PAHs (see recent reviews: Incardona & Scholz, 2017, 2018). Briefly, weathering (i.e., water‐washing of oil slicks over time) increased the proportion of 3‐ringed compounds in the water and also the cardiotoxicity (Carls *et al*. 1999; Heintz *et al*. 1999). Subsequent work in zebrafish demonstrated several key points: First, individual tricyclic compounds such as fluorene, dibenzothiophene and Phe produced a malformation syndrome (including cardiac) that was overtly similar to that from complex oil‐derived mixtures (Incardona *et al*. 2004). Second, in contrast to AhR‐mediated developmental cardiotoxicity, defects in cardiac function, observed as abnormalities in heart rate and rhythm, *preceded* the onset of heart malformation. Finally, the complex malformations observed in non‐cardiac structures (e.g. jaw defects, small eyes, body axis deformations) were all downstream of reduced cardiac output in developing embryos (Incardona *et al*. 2004). Extensive studies have now demonstrated that PAH mixtures containing nanomolar concentrations of Phe are bioconcentrated to micromolar concentrations in fish embryos (Petersen & Kristensen, 1998). Corresponding cardiotoxicity syndromes range from outright heart failure and larval mortality at the high end to subtle heart malformation with pathological hypertrophy and reduced cardiorespiratory performance at the low end (Incardona *et al*. 2004, 2005, 2009). Importantly, bradycardia and atrioventricular conduction block observed following Phe exposure in zebrafish embryos were strongly suggestive of molecular targets involving AP generation and excitation‐contraction (EC) coupling (Incardona *et al*. 2004).

In April 2010, the Deepwater Horizon disaster in the Gulf of Mexico returned global attention to the toxicity of crude oil. The blowout, the largest accidental oil spill in history, released nearly 5 million barrels of complex mixtures of PAHs into the water over a period of 87 days. Many commercially important pelagic fish species were spawning in the Gulf of Mexico during this event, including bluefin (*Thunnus thynnus*) and yellowfin (*Thunnus albacares*) tunas, and mahi‐mahi (*Coryphaena hippurus*) (Hazen *et al*. 2016). In the wake of the Exxon Valdez oil spill, damage assessment studies following Deepwater Horizon focused on the deleterious effects of oil on fish cardiac function across aquatic species, life stages and levels of biological organization (for review see Incardona & Scholz, 2018). Results showed oil‐exposed pelagic fishes also had compromised swim performance, including reductions in maximal sustained swimming speed and reduced maximal metabolic rate (Mager *et al*. 2014; Stieglitz *et al*. 2016; Nelson *et al*. 2017). Moreover, relatively short‐term whole animal aquatic exposure (24 h) to crude oil was found to reduce cardiac stroke work and cardiac output (Nelson *et al*. 2016; Cox *et al*. 2017).

### PAHs and EC coupling in fish

**Figure 3:**
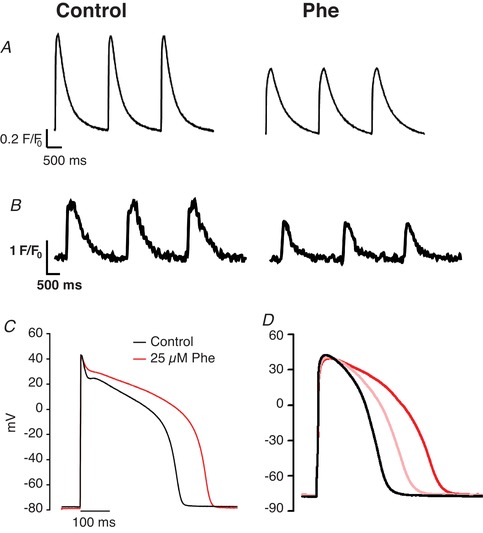
The effects of Phe on excitation contraction coupling The effect of Phe on the intracellular Ca^2+^ transient in sheep ventricular myocytes (25 µM) (*A*) and fish (bluefin tuna, 5 µM) ventricular myocytes (*B*) loaded with Fluo‐4AM and stimulated to contract at 0.5 Hz. The effect of Phe on the ventricular AP during whole‐cell current clamp in sheep (*C*) and bluefin tuna (*D*) ventricular myocytes. The red lines in both traces show data recorded during exposure to 25 µM Phe. The pink line shows the effect of 5 µM Phe exposure in tuna. No discernible effects were seen at 5 µM in sheep (not shown). Tuna data are from Brette *et al*. 2017, with permission from Scientific Reports, and sheep data are unpublished data of C.R.M., S.N.K. and H.A.S. in myocytes supplied by the members of the laboratories of Dr K. Dibb and Prof. A. Trafford at the University of Manchester.

**Figure 4:**
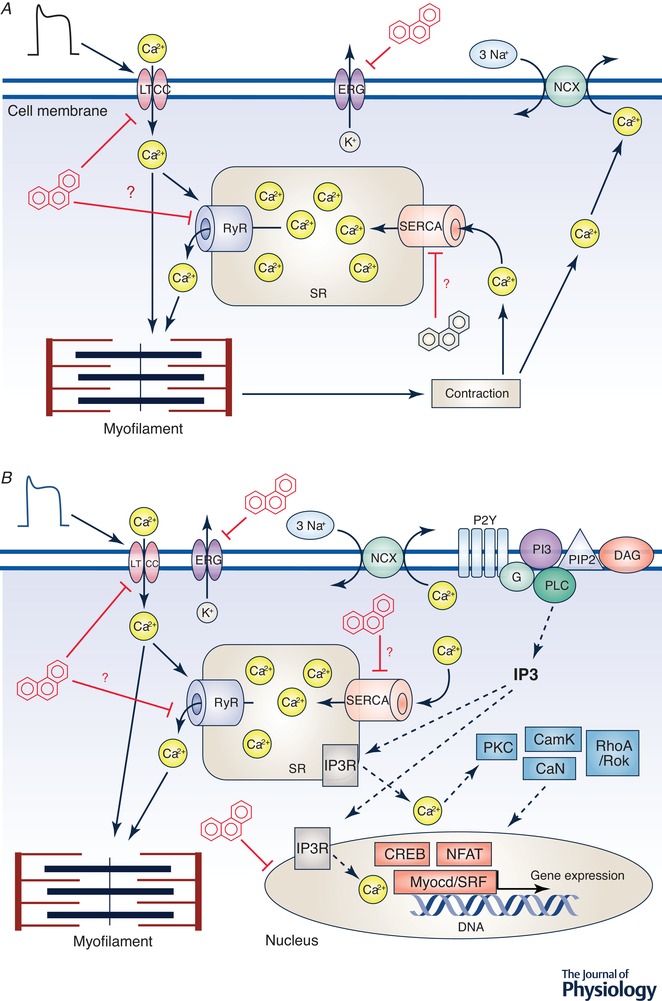
PAH and EC coupling in cardiac myocytes *A*, the top panel shows a simplified schematic of EC coupling in a cardiac cell, including the main ion channels, pumps, transporters, sarcoplasmic reticulum (SR), mitochondria and contractile proteins. EC coupling proceeds when an action potential (AP) opens L‐type Ca^2+^ channels (LTCC) in the cell membrane. This Ca^2+^ entry can directly activate the myofilament and can also initiate Ca^2+^‐induced Ca^2+^‐release from Ca^2+^ channels on SR membrane (ryanodine receptors, RyR). The global increase in the cytosolic [Ca^2+^]_i_ transient activates contractile proteins leading to contraction. Repolarization of the AP occurs in large part by K^+^ efflux through ERG (*ether‐à‐go‐go*‐related gene) channel. Relaxation follows repolarization by removal of Ca^2+^ from the cytosol primarily via re‐uptake of Ca^2+^ into the SR (via the sarcoplasmic reticulum calcium ATPase, SERCA) and extrusion of Ca^2+^ out of the cell via the Na^+^‐Ca^2+^ exchanger (NCX). Exposure to PAHs, specifically Phe, impairs EC coupling as indicated by the red inhibition bars at various ionic flux pathways. Ion flux pathways where inhibition has only been measured indirectly have a red question mark beside them. Many pathways have not yet been investigated like direct effect on myofilaments, or the NCX. *B*, interactions between EC coupling and excitation‐transcription coupling, indicating points of PAH regulation which inhibit Ca^2+^ cycling and thus transcription. EC coupling is as in *A*. Excitation‐transcription coupling proceeds when puronergic G‐protein coupled receptors (P2Y) are activated by ATP (not shown), which activates phosphoinositide 3‐kinase (PI3K) via G protein. This causes phospholipase C (PLC) to be recruited to the membrane and produce inositol 1,4,5‐trisphosphate (IP3) and diacyl glycerol (DAG). IP3 receptor‐mediated Ca^2+^ signals cause Ca^2+^ to enter the nucleus and through kinases and/or phophatases (PKC, CamK, RhoA/ROK, CaN) activate transcription factors (CREB, NFAT, Myocd/SRF) ultimately leading to gene expression. Tricyclic PAHs disrupt Ca^2+^ cycling pathways in the cell, reducing Ca^2+^ stores, and affect Ca^2+^‐related gene expression pathways. CaN, calcineurin; CamK, calcium–calmodulin‐dependent protein kinase; PKC, protein kinase C; RhoA, Ras homolog gene family, member A; ROK, rho associated kinase; NFAT, nuclear factor of activated T‐cells; SRF, serum response factor; Myocd, myocardin; CREB, cAMP response element‐binding protein.

A detailed mechanistic understanding of PAH‐induced changes in fish cardiac function came from two papers examining the effects of crude oil (Brette *et al*. 2014) and its component parts (e.g. Phe; Brette *et al*. 2017) on action potential (AP) generation and EC coupling in single isolated myocytes from pelagic fish. Consistent with the *in vivo* whole‐heart effects observed in exposed fishes, these studies demonstrated that complex mixtures of crude oil, or Phe in isolation, altered both cellular Ca^2+^ cycling and AP waveform (Brette *et al*. 2014, 2017; see Fig. 3). The studies revealed that other single PAHs (naphthalene, fluorene, carbazole, dibenzothiophene and pyrene) did not alter the amplitude or the time course of the intracellular Ca^2+^ transient in fish ventricular myocytes (Brette *et al*. 2017), and that the potency of the oil‐derived mixture was specifically correlated with the content of Phe (Brette *et al*. 2014). In addition to the depressive effects on Ca^2+^ cycling, Phe was found to affect membrane excitability by prolonging action potential duration (APD) by inhibiting K^+^ efflux from the cardiomyocyte via the rapid delayed rectifier K^+^‐current (*I*_Kr_) (Brette *et al*. 2014, 2017), analogous to the original observations in zebrafish embryos (Incardona *et al*. 2004). The depression of the Ca^2+^ transient amplitude and the AP prolongation found in bluefin tuna myocytes following Phe exposure can be seen in Fig. 3*B* and *D*. Similar effects were also found in sheep ventricular myocytes following acute Phe exposure, shown in Fig. 3*A* and *C* (Unpublished data). Recently, such disruptions to EC coupling in the myocyte have been followed through to contractile failure of cardiac tissue and abnormal contractile rhythm of the isolated whole heart in the freshwater indicator species, the brown trout (*Salmo trutta*) following acute Phe exposure (Ainerua *et al*. 2020). A summary of the known and putative effects of PAHs on cardiomyocyte EC coupling is given in Fig. 4*A*.

## Mechanistic link between PAHs and cardiovascular disease

Mammalian (including human) studies from urban areas around the world (Shah *et al*. 2015; Lee *et al*. 2018) implicate PM_2.5_ and its associated tricyclic PAHs in the induction of cardiac arrhythmias, the exacerbation of heart failure, the triggering of myocardial infarction and other atherosclerotic/ischaemic complications (Brook *et al*. 2010). Coupled with the mechanistic understanding of crude oil‐ and PAH‐induced dysfunction in the fish heart, it is certain that PAHs are pollutants of global concern. Below we have reviewed the current literature which mechanistically links PM and/or PAHs and cardiotoxicity in a range of animal models. Our aim is to show conservation of toxicant pathways and cellular targets across vertebrates to allow a broad framework of the problem to be established.

### Atherosclerosis

Atherosclerosis is a vascular disease where intraluminal fatty plaque build‐up causes a narrowing of the arteries. Rupture of unstable plaques in specific arteries can lead to thrombotic occlusion of the artery, triggering a cardiovascular event such as a heart attack or stroke. Coronary artery disease is now a leading cause of mortality in the world, affecting 2.3 million people in the UK (British Heart Foundation, 2018). A critical initiating event in the pathogenesis of atherosclerosis is endothelial cell injury. PAH uptake through inhalation puts endothelial cells at the forefront of toxic effects. BaP and other related PAHs have been found to be atherogenic, inducing vascular injury and dysfunction possibly through oxidative stress (Dhalla *et al*. 2000; Miller & Ramos, 2001). Cigarette smoke contains significant levels of Phe and smoking is a key risk factor of atherosclerosis and linked to increased endothelial cell apoptosis (Severson *et al*. 1976; Howard *et al*. 1998). Little work has been carried out on the role of individual PAHs on the apoptotic cascade (Tai *et al*. 1990) (i.e. arachidonic acid (AA) by phospholipase A_2_ (PLA_2_)); however, in 2002, Tithof *et al*. identified the apoptotic effects of three PAHs – 1‐methylanthracene, Phe and BaP – in human coronary artery endothelial cells (HCAECs) (Tithof *et al*. 2002). All three PAHs induced time‐ and concentration‐dependent release of AA, an event that precedes the onset of apoptosis. Interestingly, these PAHs activate three distinct PLA_2_ isoforms, with Phe specifically activating the Group VI and acidic calcium‐independent PLA_2_ (Tithof *et al*. 2002). A more recent study (Asweto *et al*. 2017), showed that individual PAHs could have greater toxicological effects on endothelial cells when combined with silica nanoparticles. BaP was harmless to human umbilical vascular endothelial cells (HUVECs) alone, but caused DNA damage, oxidative stress and apoptosis in the presence of particles (Asweto *et al*. 2017). These results suggest that the tissue‐specific cytotoxic effects by individual PAHs and PM warrants further study, not only in the initial stages of atherosclerosis, but also in the long‐term development and rupture of atherosclerotic lesions.

### Cardiac arrhythmias

Arrhythmias are a broad term for different types of irregular heartbeats, collectively experienced by more than 2 million people a year in the UK (National Health Service, 2018). Although often innocuous, arrhythmias can be indicative of underlying cardiac conditions and can precipitate more hazardous irregularities over time or with various stresses. There is considerable evidence showing exposure to airborne PM can affect cardiac rhythm in humans and mammalian models (Buteau & Goldberg, 2016; COMEAP, 2018). Phe exposure produces severe arrhythmias in fish, including bradycardia and AV conduction block (Incardona *et al*. 2004, 2005). For example, irregular rhythm has been observed in zebrafish embryos after 72 h of 5 nM Phe exposure (Zhang *et al*. 2013). In cats, bradycardia has been observed following high‐dose oral administration of Phe (Eddy, 1933). The mechanism of Phe action has been attributed, at least in part, to its direct influence on the multitude of ion channels involved in the AP and EC coupling (Figs 3 and 4; Brette *et al*. 2017).

Phe's effect on APD is notably detrimental. A prolongation of the APD and reduction in the Ca^2+^ transient amplitude has been observed in several fish species following Phe exposure (see Fig. 3; Brette *et al*. 2017). This prolongation of the APD is due, in large part, to an inhibition of the rapid delayer rectifier K^+^ current (*I*_Kr_), slowing K^+^ efflux during repolarization as exemplified through maximal inhibition of *I*_Kr_ by 25 µM Phe (Brette *et al*. 2017; Ainerua *et al*. 2020). Although this dose is higher than expected in plasma, tissues levels are expected to approach micromolar levels (see discussion above). This is exemplified by bradycardia (50% rate reduction) and serious fibrillation at nanomolar concentrations of Phe (0.8–0.16 µM) in the hearts of herring embryos (Incardona *et al*. 2009). Moreover, halofantrine, an antimalarial drug that has a similar structure to Phe, also inhibits *I*_Kr_ and has been associated with acquired long QT syndrome and Torsades de pointes (a specific type of arrhythmia that can lead to sudden death) (Wesche *et al*. 2000). Interestingly, recent work on rainbow trout (*Oncorhynchus mykiss*) has shown that Phe and retene (1‐methyl‐7‐isopropyl phenanthrene) shorten APD (Vehniäinen *et al*. 2019) due to varying potency of the inhibitory effect on K^+^ *versus* Ca^2+^ channels. Although AP shortening can also be arrhythmogenic, these species differences highlight the need to consider multiple species in order to understand overall PAH effects.

### EC and excitation‐transcription coupling

The reduced Ca^2+^ transients in fish myocytes following Phe exposure is attributed to an inhibition of Ca^2+^ influx through L‐type calcium channels (LTCC; see Fig. 4*A*). PAH‐induced changes in intracellular Ca^2+^ levels not only affect contractility, but may also contribute to changes in gene expression by impinging upon excitation‐transcription coupling (see Fig. 4*B*). Excitation‐transcription coupling links gene expression to the physiological state of myocytes through activation of various transcription factors like cAMP response element‐binding protein (CREB), nuclear factor of activated T‐cells (NFAT), and myocardin (Wamhoff *et al*. 2004, 2006). Perturbation of Ca^2+^ levels by Phe diminished mRNA and protein expression of the sarcoplasmic reticulum calcium ATPase (SERCA), the Ca^2+^ sequestering protein on the sarcoplasmic reticulum (SR) and T‐box 5 (Tbx5), a transcription factor regulating SERCA expression in Phe exposed zebrafish embryos and Phe exposed H9C2 cells (rat embryonic cardiac myoblasts) (Zhang *et al*. 2013).

Crude oil exposure has also been shown to alter Ca^2+^‐dependent gene expression during embryonic and early larval development in fishes including signalling pathways such as bone morphogenetic protein 10 (BMP10) and myocardin (Sørhus *et al*. 2017). The initiating event for both EC coupling and excitation‐transcription coupling are the same (Fig. 4). Electrical stimuli depolarize the membrane and trigger the opening of LTCCs and the influx of Ca^2+^, which in turn induce the cascade leading both to contraction and to Ca^2+^ regulated induction of gene expression (Fig. 4*A*) in the nucleus (Fig. 4*B*). Thus, impairment of various ionic influx pathways by PAHs may affect both excitation‐contraction and transcription‐coupling. Interestingly, Ca^2+^‐induced gene regulation appears to be sensitive to both localized Ca^2+^ increases near the site of influx and to increases in nuclear Ca^2+^ (Dolmetsch, 2003). Even though the impact on EC and excitation‐transcription coupling is reversible, downstream effects such as circulatory defects and abnormal gene expression, especially during vulnerable developmental stages, may cause irreversible morphological changes. BMP10's primary function of driving cardiac trabeculation (Grego‐bessa *et al*. 2007) was found to be up‐regulated (4‐fold) upon dysregulation of the Ca^2+^ controlled gene myocardin in response to crude oil pollutants (Sørhus *et al*. 2017). Taken together, these alterations to electrophysiology, EC coupling and gene expression in the myocyte could contribute to contractile failure, abnormal contractile rhythm and the abnormal cardiac phenotype seen *in vivo* following PAH exposure (Incardona *et al*. 2004, 2005; Hicken *et al*. 2011; Mager *et al*. 2014; Incardona & Scholz, 2016; Sørhus *et al*. 2016, 2017).

### Cardiac hypertrophy

Cardiac hypertrophy is a common pathology in many CVDs and is characterized by increased cell size and protein synthesis, ultimately leading to inefficient contractility of the heart as well as changes in coronary perfusion (Shimizu & Minamino, 2016). Cellular hypertrophy has been observed in H9C2 rat cardiomyoblasts with low Phe exposure, and in excised rat hearts where Phe increased expression of atrial natriuretic protein (ANP), brain natriuretic protein (BNP), and c‐Myc hypertrophic markers (Huang *et al*. 2016). Furthermore, DNA hypermethylation, another intracellular effect observed upon Phe exposure (Liu *et al*. 2013) has been implicated in the down‐regulation of microRNA‐133a, leading to increased expression of its target genes such as CdC42 and RhoA (involved in hypertrophic growth response) (Huang *et al*. 2016). In zebrafish, Zhang *et al*. (2013) showed that Phe exposure increased matrix metalloproteinase‐9 (MMP‐9) expression and activity. MMP‐9 is an important regulator of cardiac tissue remodelling, disruption of which has been shown to cause cardiac dysfunction such as dilated cardiomyopathy (DCM) via ventricle dilatation and wall thinning. This study also identified up‐regulation of transforming growth factor‐β (TGF‐β), a regulator of collagen synthesis. This increased activity of MMP‐9 and TGF‐β corresponds to degradation of extracellular matrix and disruption of collagen content of cardiac tissue leading to DCM (Pauschinger *et al*. 1999). Notably, these works support the idea that low levels of Phe exposure are able to change gene and protein expression in animal and cell models (see Fig. 4*B*).

Altered cardiac remodelling associated with air pollution has also been recently identified in humans. In a large asymptomatic population, with no prevalent CVD, prior exposure to PM_2.5_ (specific PAHs were not described) was associated with increased left and right ventricular end‐diastolic volume and end‐systolic volume (Aung *et al*. 2018). A comprehensive mechanistic study by Wold *et al*. (2012) further demonstrated that long‐term (9 months) PM exposure was associated with cardiac hypertrophy, changes in cardiac myocyte phenotype and impaired cardiac contractility. These demonstrate a means by which PM could induce/exacerbate heart failure and provide evidence of additional pathways underlying the associations between inhaled PM, hypertrophy, CVD and stroke (Shah *et al*. 2013).

## Interspecies disparity: the need to expand our model systems

**Figure 5:**
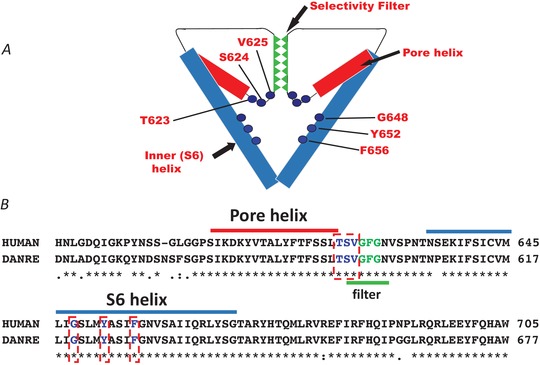
Homology of ERG channels Schematic representation (*A*) and sequence homology (*B*) of pore‐helix and S6 helix regions between hERG and zERG channels. Sequence alignments made in UniProt (Q12809 and Q8JH78). The pore‐helix is highlighted in red and S6 helix in blue. The GFG selectivity sequence is highlighted in green. Residues known to contribute to the canonical drug binding site in hERG are highlighted by red dashed boxes. Note that residue numbering in *A* refers to amino acid position in hERG. hERG:zERG equivalents: T623 = T595; S624 = S596; V625 = V597; G648 = G620; Y652 = Y624; F656 = F628.

The devastating impact of point oil spills on aquatic organisms generated significant interest in uncovering the physiological mechanisms underlying PAH toxicity. Indeed, our mechanistic understanding of PAH cardiotoxicity has been derived largely from work on fish. However, it is now well established that PAHs comprise a significant proportion of air pollution and that PAH cardiotoxicity extends to mammalian and human systems. The core functional and physiological properties of the heart are maintained across vertebrate classes, suggesting that, despite differences in routes of uptake between fish (water/gill/gut) and terrestrial mammals (air/lung/gut) (Incardona & Scholz, 2017), cardiotoxic pathways identified in fishes would be similar in other vertebrates (Shin & Fishman, 2002). Indeed, drugs that have cardiac activity in humans and other mammals have similar effects in fish (Langheinrich, 2003) and individual tricyclic PAHs cause cardiac arrhythmias in zebrafish that mirror those caused by drugs that inhibit the hERG K^+^ channel subunit (Incardona *et al*. 2004). This is not surprising given that the region encompassing the canonical binding residues between the human and zebrafish ERG differ by only a single amino acid residue (Langheinrich *et al*. 2003) (see Fig. 5). Therefore, fish, and in particular zebrafish, provide a convenient model for probing human cardiac safety pharmacology and toxicology at a functional level. Indeed, drugs that are known to prolong the QT interval in humans also modulate adult zebrafish QT interval (Tsai *et al*. 2011). In this light, it is particularly striking that an analysis of ERG blockade by over 11,000 compounds in a drug development pipeline identified peak potency associated with structures containing three aromatic rings (Ritchie & Macdonald, 2009). Thus, the wide array of Phe‐related and other tricyclic compounds associated with fossil fuel emissions are all potential contributors to acute cardiac impacts of air pollution. However, it should be acknowledged that there are also significant differences between human and (zebra) fish hearts (Table 1), particularly the molecular basis of the cardiac ionic currents which may be produced by non‐orthologous genes in zebrafish and humans (Haverinen *et al*. 2007, 2018; Hassinen *et al*. 2015; Vornanen & Hassinen, 2016). Fish also differ from mammals with respect to intracellular Ca^2+^ cycling (Shiels & Galli, 2014), again highlighting the need to study multiple species in order to understand overall PAH effects.

**Table 1:** Sequence homology in ion channels between human, hiPSC CMs and animal models

	Humana,1,2	Sheep^b^		Mouse		Zebrafish^c^		hiPSC CMs
Ion Current	Protein (Accession number)	*	Seq. homology to human (Accession no.)	*	Protein; Seq. homology to human (Accession no.)	*	Protein; Seq. homology to human (Accession no.)	*
I_CaL_	Cav1.2 (NP_955630.3)	✓ 3	92% (XP_027823851.1)	✓ 4	Cav1.2 91% (NP_033911.2)	✓ 5	Cav1.2 76% (NP_571975.1)	✓ 6,7
I_Kr_	Kv11.1 (BAA37096.1)	✓ 8	95% (XP_027824801.1)	– 9,10	Kv11.1 96% (NP_038597.2) but proscribed for repolarization assays in ICH S7B guidelines.^10^	✓ 5,11	Kv11.2^d^ 60% (NP_998002.1) 99% (canonical binding site)	✓ 6,7
I_Ks_	Kv7.1 (NP_000209.2)	✓ 8	89% (XP_027815648.1)	– 9,10	Kv7.1 88% (NP_032460.2) but proscribed for repolarization assays in ICH S7B guidelines.^10^	✓ 12	Kv7.1 67% (NP_001116714.1)	✓ 6,7
I_to_								
I_to_ (slow) I_to_ (fast) I_to_ (fast)	Kv1.4 (NP_002224.1) Kv4.2 (NP_036413.1) Kv4.3 (NP_004971.2)	✓ 13	95% (XP_027835381.1) 99% (XP_011993623.1) 99% (XP_011990111.1)	✓ 9,14	Kv1.4 97% (NP_067250.2) Kv4.2 99% (NP_062671.1) Kv4.3 99% (NP_064315.1)	– 15,16	Kv1.4 63% (XP_005169005.1) zShal2 d 80% (NP_001076336.1) zShal3 d 75% (NP_956096.1)	✓ 6,7
I_K1_	Kir2.1 (NP_000882.1) Kir2.3 (NP_690607.1)	✓ 17	99% (XP_027829809.1) 98% (XP_004023700.2)	✓ 18	Kir2.1 98% (NP_032451.1) Kir2.3 98% (NP_032453.3)	✓ 1,2,5	Kir2.4 d 67% (NP_001313403.1) Kir2.4 d 61% (NP_001313403.1)	– 6,7

hiPSC CMs, human induced pluripotent stem cell derived cardiomyocytes; * column indicating whether the human ionic current is present in the model organism: **✓** present; – negligible/not present. Numbers in brackets indicate relevant reference. Sequence homologies to humans are given in %.

^a^Ion channel isoforms most expressed in human heart. ^b^No protein name since REFSEQ is predicted by automated computational analysis. ^c^Channel isoforms most expressed in zebrafish heart and their sequence homology to corresponding human channel isoforms. ^d^Isoforms that are differentially expressed compared to humans.

^1^(Vornanen & Hassinen, 2016) ^2^(Vornanen *et al*., 2018) ^3^(Dibb *et al*., 2004) ^4^(Moosmang *et al*., 2005) ^5^(Nemtsas *et al*., 2010) ^6^(Ma *et al*., 2011) ^7^(Fermini *et al*., 2016) ^8^(Noble & Tsien, 1969) ^9^(Huang, 2017) ^10^(European Medicines Agency, 2005) ^11^(Warren *et al*., 2017) ^12^(Abramochkin *et al*., 2018) ^13^(Boyett, 1981) ^14^(Bovo *et al*., 2013) ^15^(Alday *et al*., 2014) ^16^(Nakamura & Coetzee, 2008) ^17^(Weidmann, 1955) ^18^(Lopatin & Nichols, 2001)

It is arresting that the most genetically tractable mammalian model (the mouse) has markedly abbreviated ventricular APs and despite the presence of genes for major repolarizing ion channels (see Table 1), it relies on different repolarization currents to those in humans (particularly with respect to the lack of *I*_Kr_ contribution to adult ventricular repolarization; Nerbonne *et al*. 2001; Table 1) limiting the use of the mouse for safety assurance pharmacology (European Medicines Agency, 2005). Large mammalian models and human induced pluripotent stem cell (hiPSC) derived cardiomyocytes are important platforms within drug discovery and cardiac safety testing paradigms (Fermini *et al*. 2016; Chowdhury *et al*. 2017; Sirenko *et al*. 2017) but have only very recently been utilized for PAH toxicity testing. High‐throughput *in vitro* cardiotoxicity of a library of 69 representative environmental chemicals and drugs was successfully assessed using hiPSC‐derived cardiomyocytes (Sirenko *et al*. 2017). The value of hiPSC‐derived cardiomyocytes for the evaluation of drug‐induced pro‐arrhythmia is further exemplified by the ongoing ‘CiPA’ (comprehensive *in vitro* pro‐arrhythmia assay) initiative, in which hiPSC‐derived cardiomyocytes comprise a key arm of a multi‐strand paradigm (Fermini *et al*. 2016). The translational implications of PAH research using hiPSCs in medium and high‐throughput test platforms are therefore far reaching. Our understanding of the cardiovascular effects of air pollution has been greatly expanded by using translational approaches, linking epidemiological results with cellular studies, animal models and controlled exposure in man. The addition of model fish species and high‐throughput cell assays will be valuable in identifying the action of specific PAHs on the CVS, and their interactions with other constituents within pollutants.

## Conclusions and future directions

Over the last two decades there has been considerable research into the cardiovascular effects of air pollution and many modes of inducing toxicity have been identified. However, the specific role of PAHs bound to PM requires further elucidation and the aim of this review was to stimulate investigative effort in this area. A comprehensive study of approximately 4800 tricyclic aromatic compounds in the GlaxoSmithKline drug discovery pipeline exhibited IC_50_ values ranging from 10 nM to 30 µM (mean IC_50_ ∼3 µM; Ritchie & Macdonald, 2009). Therefore, it is important to note that while Phe has been the focus of this review, other polycyclic aromatic compounds in air can synergistically contribute to cardiotoxicity. Moreover, evidence indicates that other physiological systems in addition to the CVS are affected by PAH/PM exposure, an observation that has clear parallels with the burgeoning list of extrapulmonary effects linked to air pollution over the last decade (Raftis & Miller, 2019; Schraufnagel *et al*. 2019a,*b*). Some of these pathways are acknowledged in the abstract figure which leads this article.

Due to the conserved nature of fundamental biological pathways amongst vertebrates, fish exposed to petroleum‐derived PAH mixtures in the natural environment have served as sentinels, providing significant insights into the potential human health impacts of PAHs and PM pollution. Thus, expanding research into PAH toxicity is important to fill current knowledge gaps, and strengthen the foundation for air and water policy management.

## Additional information

### Competing interests

The authors declare no competing interests.

### Author contributions

C.R.M., S.N.K. and H.A.S. drafted the manuscript with critical intellectual input from M.R.M. and J.P.I. F.B., J.C.H. and E.S. revised the manuscript and supplied specific sections. All authors contributed figures and/or text to the manuscript and approved the final version. All authors agree to be accountable for all aspects of the work in ensuring that questions related to the accuracy or integrity of any part of the work are appropriately investigated and resolved and all persons designated as authors qualify for authorship, and all those who qualify for authorship are listed. C.R.M. and S.N.K. contributed equally to this work.

### Funding

S.N.K., H.A.S., J.C.H. and F.B. (PG/17/77/33125) are supported by the British Heart Foundation, and C.R.M. is supported by the University of Manchester. E.S. is supported by Research Council of Norway (No. 267820; No. 280511). M.R.M. is supported by the British Heart Foundation (SP/15/8/31575; CH/09/002). J.P.I. is supported by NOAA National Marine Fisheries Service and NOAA National Ocean Service.
